# Exploring the Application of Peer-Assisted Learning in Practical Neuroanatomy Classes: A Cohort Comparison Within a Medical Curriculum

**DOI:** 10.1007/s40670-023-01783-2

**Published:** 2023-04-29

**Authors:** Calvin D. De Louche, Rifat Hassan, Hailey F. Laurayne, Papakas Wijeyendram, Octavia R. Kurn, James Woodward, Amgad Sbayeh, Samuel Hall, Scott Border

**Affiliations:** 1 Faculty of Medicine, University of Southampton, Southampton General Hospital, Southampton, UK; 2 Centre for Learning Anatomical Sciences, Population Sciences and Medical Education, University of Southampton, Southampton General Hospital, Primary Care, Southampton, UK; 3 School of Medicine, Dentistry and Nursing, Thomson Building, University of Glasgow, Glasgow, UK

**Keywords:** Near-peer teaching, Neuroanatomy, Medical education, Anatomy education, Co-teaching

## Abstract

Despite well-documented benefits, the effectiveness of some aspects of near-peer (NP) teaching in medical education within anatomy curricula remains unclear. Here, we explored the impact of various permutations of staff/student laboratory-based co-teaching in neuroanatomy by determining the optimal staff and student teaching combination. We assessed student perceptions and knowledge acquisition using three different co-teaching strategies. Second-year medical students at the University of Southampton were co-taught neuroanatomy by faculty staff and third-year medical students (NP teachers). Three cohorts, 2016/2017, 2017/2018, and 2018/2019, were included in the study. Subsequent cohorts experienced increasingly structured NP teaching with more NP teachers. Students completed evaluations for anatomy sessions, which were statistically compared. The 2017/2018 and 2018/2019 cohorts completed lunchtime quizzes matched to the learning outcomes of each practical session, which were analysed. A focus group involving six students was transcribed and thematically analysed. Anatomy practical ratings were significantly higher when both session structure and NP teacher numbers increased from 3 to 5–6 (*p* = 0.0010) and from 3 to 7–8 (*p* = 0.0020). There were no significant differences in anatomy practical ratings using 5–6 and 7–8 NP teachers (*p* > 0.9999). There were no significant differences between the knowledge scores of students who experienced 5–6 and 7–8 NP teachers. Themes detailing the benefits of NP teaching and the importance of faculty involvement were identified, demonstrating that students appreciated NP teaching within a co-teaching environment. Therefore, increased NP teaching may augment students’ perceptions and knowledge acquisition. In this context, the optimal number of NP teachers may sit between 5 and 8.

## Introduction

### An Introduction to Near-Peer (NP) Teaching

Whitman first introduced the term near-peer (NP) teaching in the late 1980s [1]. Despite discrepancies surrounding the definition of NP teaching, it can be broadly defined as the phenomenon whereby senior trainees (one or more years senior in training and on the same level of the education spectrum) teach more junior trainees [2]. The methods described have since been used in various subject areas, including environmental education, language teaching, engineering, and mathematics [3–8]. NP teaching also has widespread applications in the context of medical education and can be used to teach clinical skills, technical skills, and gross anatomy in the laboratory [9–11].

In the last decade, there has been a greater uptake of NP teaching in medical education, due its perceived benefits for student learners and student teachers [12, 13]. NP teaching has been evidenced to enhance the learning experience for the student learner [14]. This may incentivise medical schools to implement NP teaching to achieve greater student satisfaction rates. This is particularly pertinent in the UK, where institutions may seek to implement NP teaching to achieve higher ratings within the Teaching Excellence Framework [15] and on the National Student Survey [16]. As both measures contribute to a medical school’s national reputation, implementing NP teaching may serve as a mutually beneficial approach for both the student learner and their institution.

#### Benefits to the Student Learner

NP teachers and student learners have a similar knowledge or ‘cognitive congruence’. This allows NP teachers to use language and prosody of speech that student learners can better comprehend. This cognitive congruence depends on the teacher’s expertise and the social congruence between teacher and student learner [17]. Due to recent experiences with course content, NP teachers are more likely to be familiar with the struggles of the student learners. They can explain known threshold concepts at a more accessible level when compared to faculty staff [17, 18]. NP teachers and student learners also share similar social congruences, which permits NP teachers to understand better the student learner’s academic and social lives [17]. This allows NP teachers to promote a more comfortable learning environment where student learners can freely express themselves and fully participate in the learning [19]. We have previously explored the role of congruence in NP teaching, demonstrating that senior medical students were rated higher in the criteria for cognitive and social congruences when compared with their junior doctor counterparts [20]. Therefore, NP teachers sharing similar congruences with younger student learners may generate a more effective learning environment when compared to their more senior colleagues.

NP teachers may have less knowledge and experience when compared with faculty staff. However, this does not necessarily negatively impact the student learners. It has been reported that student learners recognise that NP teachers take more time to explain concepts when compared to faculty staff, which provides more opportunities to raise questions. Additionally, this extra time was beneficial in assisting the learners to process new concepts [2]. More importantly, the literature demonstrates that stand-alone NP teaching leads to the same levels of success in achieving the learning outcomes and, in some cases, even surpasses like-for-like faculty teaching [21–23]. Student learners also feel NP teachers give better quality feedback [24], which may allow them to better understand their current knowledge surrounding which topics/concepts to improve to achieve better assessment results.

#### Benefits to the NP Teacher

Teaching is an essential skill a doctor must possess. As such, medical schools must train students to ensure they are adequately prepared [2]. Additionally, the General Medical Council mandates doctors to be ‘familiar with a range of teaching and learning techniques’ and ‘recognise their obligation to teach colleagues’ [25]. Fundamentally, teaching skills are acquired through training, practice and feedback, which presents NP teaching as a prime avenue for medical schools to provide such opportunities to their students [26].

The literature is well-populated with evidence to support the benefits of partaking in NP teaching for the NP teacher. Evans and Cuffe reported that NP teachers felt their general oral communication skills had improved by participating in NP teaching programmes and subsequently that their career development had been augmented [27]. This highlights the range of transferable and professional skills that NP teachers can enhance within NP teaching programmes and use in their future careers as doctors [28]. Moreover, Ten Cate and Durning found that teaching others promotes deeper learning for the teacher, as it increases their motivation to learn the content thoroughly [29]. Subsequently, they reported that teachers may have better knowledge retention and an improved grasp of the subject they teach [29]. This is reinforced by further work suggesting that student teachers attain greater cognitive gains whilst teaching when compared to student learners during NP teaching sessions [30].

### Deploying NP Teaching in Neuroanatomical Education

Anatomy embodies one of the fundamental pillars of medical education, as every medical specialist requires detailed anatomical knowledge of their specific field [31, 32]. However, the current decline in the perceived importance of, and funding for, anatomical teaching in UK medical schools poses a serious threat to the future of patient care [31–34]. Many doctors feel they lack sufficient anatomical knowledge when graduating from medical school. This could be due to a decrease in the allocation of both pre-clinical hours for teaching and time spent in the laboratory in exchange for more professionalism-based content, but also that many institutions have shifted away from full body dissection in favour of prosections and technology-enhanced learning strategies. Subsequently, many now emphasise the importance of improving anatomical teaching within medical schools, indicating its importance in patient care [35].

Within anatomy, there exists a common fear amongst students with neuroanatomy specifically, as it is perceived to be more complex than other anatomy modules [36, 37]. To address this, the University of Southampton implemented an NP teaching programme in their ‘Nervous and Locomotor 2’ (NLM2) module for medical students, covering anatomy practicals and clinical neuroanatomy sessions. We have previously reported that students being taught as part of this programme felt more confident and had significantly increased perceived levels of knowledge after attending NP teacher-led clinical neuroanatomy sessions [38]. Thus, NP teaching may lead to positive learning outcomes in subjects traditionally perceived as difficult in medical school, such as neuroanatomy [38]. The opportunity to teach in a laboratory-based setting is also often considered highly desirable by student teachers who sign up for the programme. Here, they choose an environment where they co-teach alongside anatomists (using prosected cadaver resources) rather than lead a standalone NP teaching session.

### Why Co-Teaching?

Co-teaching is when ‘two or more professionals deliver substantive teaching to a diverse or blended group of students in a single physical space’ [39]. At the University of Southampton, student teachers receive mandatory training and work closely with staff using a partnership approach [40, 41]. This may lead to student learners perceiving student teachers as staff members and subsequently alter the dynamics of NP teaching. For example, it could potentially affect the cognitive congruences that made NP teaching such an effective tool. Hence, this proposes a further reason to investigate the utility of this staff/student co-teaching environment.

Existing evidence has demonstrated that co-teaching significantly increased knowledge retention compared to conventional teaching methods within an integrated medical curriculum. However, the study found no significant differences in the learning outcomes between the two methods [42]. Given the success of NP teaching, it appears worthwhile to explore the effectiveness of faculty staff and NP teachers co-teaching together; this dynamic may enhance the learning experience. The co-teaching relationship could aid with assisting and advising the faculty when planning anatomy sessions by incorporating the input of NP teachers. This may enhance the student learner’s engagement with the learning material and reduce faculty/student communication barriers that may have existed beforehand [43, 44].

Current evidence suggests that students appreciate the increase in the accessibility of teachers with the integration of NP teaching and that they found NP teachers to be a reliable source of knowledge [45]. This could prove helpful in anatomy laboratory environments, where faculty staff may often be occupied when dealing with a high number of student queries. Moreover, it also addresses the notion that students tend to rate the effectiveness of teachers lower when the class sizes are larger [46]. Therefore, co-teaching may promote a more efficient, inclusive learning environment whereby students can approach teachers with queries more readily due to an increase in their availability. This raises the question of how NP teachers would be perceived when co-teaching alongside anatomist staff in the lab. It could be speculated that attitudes towards their role might differ compared to standalone NP teaching sessions. If learners accept and treat them more like staff, there could be a risk that some of the congruence benefits could be compromised.

### Co-Teaching in Neuroanatomy

Despite the current literature overwhelmingly supporting the use of NP teachers in neuroanatomical education to co-teach alongside faculty staff, there remains a lack of research investigating the most effective implementation of NP teaching. Notably, a gradual increase in the numbers of NP teachers teaching in a neuroanatomy course across 5 years was positively correlated with improved student perceptions, where, as the numbers of NP teachers increased, students felt that there was an increase in positive contribution to their learning and their motivation to find answers to questions posed by them [47]. Despite these findings, there remains a relative paucity of data surrounding the effects on student learners’ knowledge alongside their learning experiences in relation to an increase in the implementation of NP teaching within a co-teaching environment. In our study, we aimed to contribute to this knowledge base by evaluating student perceptions and impact on knowledge acquisition when implementing differing co-teaching approaches within the neuroanatomy course at the University of Southampton over 3 years.

## Materials and Methods

### Ethics Statement

Ethical approval was obtained from the Faculty of Medicine Ethics Committee, University of Southampton, before commencement. This approval was filed under Ethics and Research Governance Online ID 49877.

### Practical Teaching Structure and Participants

Within the medical programmes at the University of Southampton, neuroanatomy and head and neck anatomy are combined within a systems-based module called Nervous and Locomotor 2 (NLM2). This is a second-year module which focuses on the central nervous system. The Nervous and Locomotor 1 module (NLM1) is taught in the first year and covers the peripheral nervous system and musculoskeletal anatomy; it provides the prerequisite knowledge for NLM2. In NLM2, students receive 10 hours of practical/workshop teaching and 10 hours of e-learning activities that support the same learning outcomes. The anatomy lead for the module delivered 11 lectures of 45 minutes throughout the 8-week module.

Three cohorts of 2nd-year undergraduate medical students at the University of Southampton were involved in this mixed-methods study. The 2016/2017 cohort contained 210 students. The 2017/2018 cohort contained 228 students, and the 2018/2019 cohort contained 215 students. In each cohort, students were split into 3 laboratory teaching groups (A, B, and C).

There were 4 practical anatomy sessions lasting 2 hours, each containing 8 individual stations for students to study. The purpose of each station was to teach a specific set of 2 or 3 core learning objectives regarding a particular area of the head and neck/neuroanatomy curriculum. For instance, one station may have covered the arterial supply of the head and neck (e.g. branches of the external carotid artery) whereas another could, for instance, have focussed on the muscles of mastication. Learning objectives from these stations were linked to the content being taught and primarily centred on identification (i.e. can the student identify structure X), function (what does structure X do), and application (how does the function of structure X relate clinically) style outcomes. Eight similarly themed stations were grouped together to form a full anatomy practical session. Each individual station lasted for 15 minutes. Students were given a workbook with activities and questions at each station and encouraged to work in small groups. These were solely provided as a learning guide and were not mandatory to complete. The beginning of each practical session was always introduced by a member of faculty staff, who demonstrated some core anatomy components of the practical to help students get started. After this, staff and NP teachers co-taught for the remainder of the session.

The arrangement of these practical sessions evolved iteratively with each subsequent cohort to become more structured. Adding structure refers to the stricter management of time and support at the station level through the increased number of NP teachers per session (Table 1). These changes came via the evolution of our ongoing investigations into NP teaching efficacy and a growing demand for NP teaching opportunities from senior medical students. The combination of head and neck anatomy and neuroanatomy together plus a reduction in curriculum time from 13 weeks in 2007 to 8 weeks in 2019 gave this module a reputation as being the most difficult of the pre-clinical phase. Feedback from the practical sessions had suggested that there were not enough staff on hand to guide students through the prosections at each station and that there was overcrowding around certain specimen tables, suggesting that the learning experience was not equitable. Deploying increasing numbers of trained NP teachers and adding structure to the practical sessions was used to remedy some of these problems.

**Table 1:** Implementations of NP teaching in the three cohorts

**Cohort**	**Number of NP teachers per anatomy practical session**	**Approach**	**Time management**
2016/2017	3	Self-directed	Self-directed
2017/2018	5–6	Semi-structured	Self-directed but with notifications
2018/2019	7–8	Structured	A countdown and buzzer that signalled a change of station

In the current study, NP teachers were 3rd-year undergraduate medical students at the University of Southampton who had previously studied and passed the NLM2 module. They had been carefully selected using non-academic criteria (predominantly centring on their commitment to attending training sessions, reliability, and ability to plan/organise), rigorously trained and deemed competent by faculty staff members to teach the required material. Their involvement in the NP teaching programme was entirely voluntary. NP teachers delivered NP teaching in the weekly anatomy practical sessions with varied approaches for each cohort. NP teachers only taught the content of their specific station.

The 2016/2017 cohort contained the lowest number of NP teachers per practical session. Here, NP teachers undertook more of a facilitatory role, whereas student learners took a self-directed approach and managed their own time between stations. In contrast, NP teachers in the 2017/2018 cohort had more involvement in leading sessions, allowing them to take responsibility for delivering learning outcomes over a range of practical stations. Student learners in this cohort were advised by the anatomy tutor when to move on to a new station so that they could cover all the learning outcomes during the allotted time. The 2018/2019 cohort experienced this study’s most tightly structured setting. Every practical anatomy station was manned by an individual NP teacher who delivered precise, pre-prepared teaching. Even groups of student learners visited each station for a pre-specified amount of time before rotating around the laboratory to the next station. In all NP teaching approaches, 2 members of laboratory staff oversaw the session, but they did not actively participate; they only answered questions when passed on by NP teachers.

The content covered in all 3 years was fundamentally the same and was guided by the same learning outcomes. Additionally, the workbooks covering the core content given to each student remained identical in all 3 cohorts, irrespective of the iteration of NP teaching that they received. During the study timeline, there were no significant curricula revalidations or amendments to the anatomy module’s learning objectives. Students in all cohorts were encouraged to supplement whatever they had learnt in the NP teaching sessions with private study. This refers to the students visiting the lab in their own time, whilst the practical was set up, to work at their own pace through the stations.

### End of Module Evaluations

University of Southampton medical students were asked to provide a satisfaction rating at the end of each system-based module during their first 2 years of study (using a 5-point Likert scale). This was a standardised, anonymous, formal module evaluation process distributed to all students. We used this module evaluation data to assess student learners’ perceptions of the varied implementations of co-teaching.

All 3 cohorts were asked, ‘Overall, how would you rate the following non-lecture sessions?’. The ‘non-lecture sessions’ data looked at student learners’ responses to ‘Anatomy Practical Sessions’ only, as these were the only ‘non-lecture’ sessions in which NP teaching was implemented. Non-lecture sessions without NP teaching were excluded from analysis. The options by which student learners could respond ranged from ‘Poor’ to ‘Excellent’. Data was gathered and recorded in Microsoft Excel (Microsoft Corporation, 2018). For data analysis purposes, student ratings were cleaned and converted to a numerical scale (i.e. ‘Poor’ = 1, ‘Excellent’ = 5). All data from the 3 cohorts were inputted into SPSS Statistics 26 (IBM, 2018). The Kruskal–Wallis test was used to analyse the quantitative data since it was deemed to be non-parametric (Shapiro–Wilk, *p* < 0.05) and involved a comparison of more than 2 groups.

### Focus Group

Focus groups were conducted to gather qualitative insights to triangulate with the quantitative data from module evaluations. This would allow us to further analyse student perceptions with the different implementations of NP teaching. Third and fourth-year medical students who had experienced NP teaching during the second year of their course were invited to participate via social media and recruitment posters. Six interested students participated in the focus group. All were part of the 2018/2019 cohort. Before the focus group discussion, all interested participants were emailed a participant information sheet and were able to ask any related questions or opt out at any stage. On the day, participants were provided with a consent form to sign if they wished to participate. Afterwards, the group of students and the primary author sat in a room together. A brief introduction was provided explaining the purpose of the session. A list of prompts was then worked through to gather student views on NP teaching. These included questions such as ‘What are your experiences of NP teaching in the NLM2 module at medical school?’, ‘What do you think the benefits/drawbacks of NP teaching are?’, ‘What are your thoughts on students and staff teaching together’ and ‘How credible do you think NP teaching is?’, amongst others. The focus group discussion lasted 90 minutes and was recorded and transcribed by a university-approved professional transcription service. The transcription was thematically analysed using NVivo 12 software (QSR International, 2018).

### Lunchtime Quizzes

All second-year medical students were invited to voluntary lunchtime quizzes during their NLM2 module. This was an optional session that consisted of self-selecting students only. Throughout the module, willing participants answered a total of 80 questions. This was broken down over 4 separate lunchtime quiz sessions containing 10 questions. Each of these 10 questions had a part A and part B. Both parts of the question were worth 2 marks (Appendix A). Questions were shown on screen for 1 minute in the anatomy laboratory (to mimic the time students would receive in their summative examination) and aimed to test both the identification and application of knowledge of anatomical concepts. The questions were based on the learning outcomes of the previous week’s anatomy practical sessions and therefore assessed the student’s short-term knowledge acquisition. Students recorded their answers on a pre-prepared, anonymous answer sheet. Scores from these sessions did not form any part of a summative course grade, but instead allowed students to assess their knowledge in an informal, non-graded format. Data from the lunchtime quizzes were gathered from the 2017/2018 and 2018/2019 cohorts only.

An independent *t* test was used to compare the knowledge scores of the 2 cohorts as the data was deemed parametric (Shapiro–Wilk, *p* > 0.05). Additionally, Levene’s test for the equality of variances was performed. Cohen’s *d* test was also carried out to investigate the effect size of any differences. For all statistical tests in this study, *p* < 0.05 was accepted as a marker of statistical significance.

## Results

### Evaluation of Student Perceptions

#### End of Module Evaluations

All students in each cohort were requested to complete an end-of-module evaluation form. Individual responses alongside the overall response rate are shown in Table 2. A Kruskal–Wallis test revealed the distribution of ratings between the 3 cohorts to be significantly different (*p* = 0.0010). The Dunn’s multiple comparison post hoc test was used to determine the detail and nature of the significance between individual cohorts. The student ratings of the anatomy practical sessions between the end of module evaluations in the 2016/2017 to 2017/2018 cohorts (*p* = 0.0010) and the 2016/2017 to 2018/2019 cohorts (*p* = 0.0020) were significantly different. The 2017/2018 and 2018/2019 cohorts did not provide scores that were significantly different to each other (*p* > 0.9999). Moreover, the median rating given by the 2016/2017 cohort was 4 (Good), compared to 5 (Excellent) for the 2017/2018 and 2018/2019 cohorts. Taken together, these data highlight that students in the 2017/2018 and 2018/2019 cohorts gave higher ratings on average when compared to the 2016/2017 cohort.

**Table 2:** End-of-module Likert scale ratings in each cohort, expressed as a percentage of the total number of responses

**Cohort**	**Poor (%)**	**Below Average (%)**	**Average (%)**	**Good (%)**	**Excellent (%)**	**Total Responses (n)**	**Response Rate (%)**
2016/2017	1	7	17	42	33	143	68
2017/2018	1	2	8	39	50	131	57
2018/2019	2	2	8	37	51	104	48

### Focus Group

Thematic analysis of the focus group transcription generated 72 individual codes. Four categories were identified (Table 3). A sample of representative quotes from participants regarding all categories and themes is included in Appendix B.

**Table 3:** Categories identified from thematic analysis and the corresponding number of codes for each category

**Categories**	**Number of codes**
Benefits of NP teaching	36
Credibility of NP teaching	5
Implementing NP teaching in other anatomy modules	13
Importance of faculty involvement	18
**Total**	**72**

### Benefits of NP Teaching

The category ‘Benefits of NP teaching’ generated 36 codes, of which 31 codes formed the theme ‘Benefits to Student Learners’ (Table 4).

**Table 4:** Themes assigned to the ‘Benefits of NP teaching’ category

Category	Theme (codes)
Benefits of NP teaching	Benefits to student learners (31)
	Benefits to student teachers (3)
	Benefits to the faculty (2)

Overall, all participants felt NP teachers were more approachable than faculty staff and could teach competently. Most participants highlighted that the variety of teaching styles that NP teachers provided made learning the content easier and more concise. In addition, most participants felt privileged to access online resources produced by NP teachers and thought that this significantly contributed to their learning. They also appreciated the out-of-hour teaching sessions provided by NP teachers, which helped summarise key topics within the curriculum. They felt that both would have been hard to implement without the use of NP teachers and, as such, were highly effective educational tools. Participants also believed that NP teachers could gain skills from teaching that may be useful. Moreover, they felt that NP teachers reduced the pressure on the faculty, allowing faculty staff more time to facilitate sessions more effectively.

### Credibility of NP Teaching

Five codes were related to the ‘Credibility of NP teachers’ (Table 5).

**Table 5:** Themes assigned to the ‘Credibility of NP teachers’ category

**Category**	**Themes (codes)**
Credibility of NP teachers	Less trust in NP teachers (4)
	NP teachers taken less seriously (1)

Some participants felt that they could not fully trust the NP teacher’s knowledge and, as such, could not fully be sure of the quality of their teaching compared to a professional anatomist.

### Implementing NP Teaching in Other Modules

Many of the themes in this category were identified from the question ‘Do you think co-teaching sessions are more or less useful when compared to exclusively faculty-led practical sessions?’. Thirteen codes were identified (Table 6).

**Table 6:** Themes assigned to the ‘Implementing NP teaching in other modules’ category

**Category**	**Themes (codes)**
Implementing NP teaching in other modules	Importance of independent learning (10)
	Lack of teaching in other anatomy modules (2)
	Incorrect learning in other anatomy modules (1)

One participant did not have the best experience with NP teaching within NLM2 anatomy practicals. They preferred self-study methods implemented in the other anatomy modules, whereby they only referred to faculty staff whenever they found a topic extremely difficult. This opinion may have influenced the views of others, which led to most of the participants highlighting the importance of independent learning and the importance of finding a balance between learning from teachers and independent learning.

Most participants felt that the self-study approaches in the other anatomy modules negatively impacted their learning. This was because there were far fewer available teachers, so it was impossible for the teachers to teach all students optimally. Therefore, most students felt it increased their chances of learning anatomy incorrectly, as other modules consisted of mainly independent learning and nowhere near enough tutor-led support.

### Importance of Faculty Involvement

Of the 18 codes relating to the importance of faculty involvement, 15 related to the availability of staff to help and provide reassurance by their presence to both the student learners and teachers (Table 7).

**Table 7:** Themes assigned to the ‘Importance of faculty involvement’ category

**Category**	**Themes (codes)**
Importance of faculty involvement	Available to help (15)
	Reassuring presence (1)
	Student preference (1)
	Accredit teaching of NP teachers (1)

Participants felt that if faculty staff were not present, student learners would have less trust in NP teachers. Also, the participants noted that sometimes NP teachers could not answer some of their questions. Therefore, NP teachers relied upon the presence of faculty staff to assist in those situations. Participants felt that the faculty staff are essential in ensuring that NP teachers are teaching correctly and up to standard, highlighting that the faculty still have a significant role in facilitating the NP teaching co-teaching sessions. Also, one participant expressed that they prefer to be taught mainly by faculty staff, not NP teachers. This was because they had paid considerable money to study at university and be taught by experienced teachers. Therefore, they would like to receive the tuition they are paying for.

### Evaluation of Student Knowledge

#### Lunchtime Quizzes

Participants answered a total of 80 questions. This was broken down over 4 separate lunchtime quiz sessions containing 10 questions. Each of these 10 questions had a part A and part B. Both parts of the question were worth 2 marks. The mean lunchtime quiz score per question from the 2017/2018 cohort was M = 1.006 (SD = 0.23) compared to M = 0.955 (SD = 0.24) for the 2018/2019 cohort. The independent *t* test comparing scores between cohorts was associated with no significant differences (*p* = 0.883). Moreover, Levene’s test highlighted the homogeneity of the variances (*p* = 0.625). In summary, the two cohorts had no significant differences in knowledge acquisition. Moreover, Cohen’s *d* was 0.0480, denoting the effect size of the differences between the cohort’s quiz scores to be very small.

## Discussion

### Our Findings

Continued reductions in anatomy teaching posts have led some anatomists to believe that the provision of anatomical education in medical schools is still experiencing a ‘downward spiral’ [48, 49]. Therefore, incorporating NP teaching with a co-teaching structure or as a standalone entity may allow anatomy departments to combat the restraints they currently face and simultaneously augment the experience and knowledge acquisition of student learners [11, 14, 27].

Our quantitative findings suggest that, generally, an increase in the number of NP teachers within neuroanatomical laboratory-based teaching results improves the perceptions of student learners. These findings corroborate the results of previous work reporting a positive correlation between student learner perceptions and increased implementation of NP teaching [47]. We must, however, acknowledge the potential influence that the added structure of these sessions with more NP teachers may have had on improving learner perceptions. In our study, student anatomy practical ratings were rated significantly higher when the number of NP teachers was increased from 3 to 5–6 and from 3 to 7–8. We observed no significant difference between student anatomy practical ratings using 5–6 and 7–8 NP teachers. This was strengthened by the findings that there were no significant differences between the mean knowledge scores of the students between the two cohorts that experienced 5–6 and 7–8 NP teachers, respectively. These results suggest that the optimal number of NP teachers may be any number between 5 and 8 for an 8-station practical. This range may act as an effective number of NP teachers to deploy within an anatomy class at a medical school with a similar class size of circa 70 students. In broader terms, our data suggest that it may be more effective to incorporate higher numbers of NP teachers using a more structured NP teaching approach in a neuroanatomy course within the co-teaching environment.

The 2016/2017 cohort experienced a self-study learning approach that used NP teachers as facilitators rather than tutors. This reflects the historical values of the Faculty of Medicine at the University of Southampton and its broad alignment to the principles of a constructivist approach within undergraduate anatomical education. The theory of constructivism encourages student learners to link and adapt their previous knowledge to stimulate learning new knowledge [50]. Despite this, 5 of the 6 focus group participants highlighted that in other anatomy modules with no NP teachers and fewer available tutors, they were more likely to learn the content incorrectly, as they felt they did not receive enough tutor-led support to learn by themselves confidently. There is, of course, a proclivity towards determining the right balance between tutor-led support and independent learning since opportunities for the student learners to undertake self-study are compromised by the length of time the curriculum allows them to learn the material [48]. Striking this balance may be crucial and is, in part, interconnected with the principles of cognitive theory. The concept of applying cognitivism to augment learning processes has existed for decades and is well described [51, 52]. Here, using cognitivism principles ensures that student learners are not too far removed from their learning experiences [51, 52]. This could work well in the context of NP teaching in a co-teaching environment, as student learners will likely have more capacity to integrate NP teaching and faculty staff instruction. Additionally, student learners may be more likely to promote self-testing as another method by which they can learn formatively rather than as an assessment of knowledge.

### Determining the Optimal Number of NP Teachers

Not only will student learners benefit from the increase in the availability of teachers by incorporating as many NP teachers as possible, but also higher numbers of senior medical students may benefit from teaching others [53]. More NP teachers being deployed to deliver content increases the opportunities for medical students to enhance their teaching skills and gain a deeper understanding of anatomy. These aspects will likely benefit their future careers as practicing physicians [27–29]. Whilst medical schools retain a certain degree of flexibility over the numbers of students that can teach on new NP teaching programmes, our findings suggest that the implementation of a greater number of NP teachers provides superior outcomes and, thus, may be required to return optimal educational benefits for both NP teachers and student learners alike.

Overarchingly, our data indicate that the use of 5 NP teachers in an 8-station practical may be the most effective. The current study demonstrates that deploying more than 5 NP teachers has no added benefit in augmenting student perceptions and knowledge acquisition in this context. In fact, from an observational perspective, it could be speculated that a surplus of NP teachers leads to a passive learning environment where learners have limited time for self-exploration, which would be detrimental to aiding a deeper understanding of the subject matter. It stands to reason that student learners prefer to have more NP teachers than fewer if offered the choice. As a result, we theorise that increased NP teaching availability may allow for combining various complementary pedagogical resources that act synergistically to provide students with constant support during live teaching. This may include, but is not limited to, the integration of multimodal, system-based approaches that students have previously shown to benefit from [54, 55].

### Does Class Size Matter?

Existing evidence demonstrates that NP teachers can stimulate effective learning due to their ability to facilitate small teaching groups within the anatomy practicals [56]. Critically, this is unlikely to be feasible if the number of NP teachers was too low. This is reiterated by the findings from our focus group, where many participants highlighted the problem of the laboratory being crowded with students, regardless of the high numbers of NP teachers. Some participants alluded to a need to reduce the number of students within the anatomy practical sessions to gain the benefits of NP teaching fully. This comment may also apply to teaching in general without the use of NP teachers. However, in this regard, there remains contention in the literature, as a reduction in class size has been evidenced as not always being the most effective way of improving student learning outcomes [57–59]. An improved student learning experience will partly depend on class size, as evidence suggests that a greater ratio of staff to students leads to better student satisfaction [60, 61]. Thus, institutions may seek to target these strategic levers to enhance their outcomes from the National Student Survey, which feeds directly into the Teaching Excellence Framework [15, 16].

### Impact of Co-Teaching

It has been reported that when students and faculty co-teach together, an environment is created that improves the overall learning experience of student learners [45] and their performance [62]. Interestingly, in our focus group, one participant warned that we should be careful about the current emphasis on greater implementation of NP teaching. This is because faculty members may be capable of promoting alternative and deeper approaches to learning. Moreover, faculty staff could use their invaluable experiences and apply these to teaching the principles of anatomy. It was emphasised how the presence of faculty staff provides reassurance and confidence in NP teachers. This suggests that students may not find stand-alone NP teaching as trustworthy or effective and would thus prefer medical schools always to deploy NP teachers in a co-teaching environment (i.e. something analogous to the NP teaching programme outlined in this paper).

In conrast, other reports in the literature indicate that stand-alone NP teaching is as effective, if not more effective, compared to the faculty-led teaching [21–23]. Sharma and colleagues had previously demonstrated that co-teaching methods increased knowledge retention compared to conventional standalone teaching methods. However, they too did not find a significant difference between student learning outcomes using both methods [42]. Thus, it is evident that there remains a paucity of data that compares the effectiveness of standalone NP teaching with a co-teaching model concerning long-term student outcomes. Ideally, this is something that future research could seek to address. In this regard, the authors recommend work that scrutinises objective outcome measures incorporating coordinated knowledge retention tests using both approaches.

Undoubtedly, co-teaching approaches are more beneficial to the faculty than standalone NP teaching. We posit that the benefits of using a co-teaching approach primarily lie in the similar cognitive and social congruences that NP teachers share with student learners [17, 18, 63]. This, in turn, may enhance the student learner’s engagement with the material and subsequently reduce the communication barrier that would have likely existed between students and staff in the absence of co-teaching [43]. These benefits would fail to exist outside of the co-teaching model proposed here.

Despite this, comments from our focus group regarding the relationship with and behaviour of NP teachers allow us to theorise that NP teachers may not be as effective in the absence of faculty staff. Although there are very few published reports on such a dynamic, our findings suggest that there is, perhaps, a conflicting argument to be made. This centres around the idea that similar social congruences between teacher and learner, in the absence of faculty staff, might not be as valuable as first thought as it has the potential to lead to an off-topic conversation that would likely hinder learning. This permits us to tentatively postulate that in the absence of faculty staff and owing to their similar social congruence to their student learner, NP teachers may be less effective in delivering the required learning outcomes. It is important to mention that this hypothesis contradicts the vast body of literature that supports the benefits of similar social congruences between teacher and learner [17–20, 63–65]. Additionally, a logical argument suggests that because NP teachers work alongside staff, they may be treated more like they were staff. Thus, we may speculate that if learners no longer treat NP teachers like peers, many benefits of social and cognitive congruence could potentially be lost. Although this constitutes a reasonable hypothesis, there is currently no empirical evidence to support it. Further studies would be required to explore the impact of social congruence between teacher and learner on learning outcomes in a co-teaching environment to provide a firmer basis for these suppositions.

### Limitations

Although we believe our study to have many merits, we must address its limitations. One important limitation was that we could not record data detailing the lunchtime quiz scores for the 2016/2017 cohort due to the timeline of ethical approval. This may have provided evidence for, or against, the use of 5–8 NP teachers. Our study was a longitudinal cohort study, so it was difficult to deploy experimental designs within a strictly controlled curriculum environment [66]. Similarly, no data is recorded for a control cohort without NP teachers.

Additionally, there is a chance that the end-of-module evaluation questions may have been perceived as non-specific, primarily as they were not intended to inform the study. The question asked students how they would rate their practical anatomy sessions but did not reference the input of NP teachers specifically. The response rate to these evaluation forms was also relatively low, especially in the 2018/2019 cohort, which may have made it more difficult to conclusively determine student satisfaction. Despite this, the data was still valuable for the benefit of multiple cohort comparisons.

Moreover, our focus group was entirely self-selected, meaning the results are less likely to accurately reflect the views of the entire cohort [67]. Additionally, all focus group participants were part of the 2018/2019 cohort, which had experienced the structured approach of NP teaching. Therefore, there may have been some elements of bias involved, as they had only experienced one of the three possible NP teaching implementations that being the most structured. Finally, as this was a single-centre study, the generalisability of our findings is limited beyond the immediate context.

## Concluding Remarks

In summary, we have explored the teaching dynamics of utilising NP teachers in a staff/student co-teaching model during neuroanatomy laboratory practical classes within a medical school setting. A combined discussion of qualitative and quantitative analyses reveal that most student learners would prefer co-teaching applications of NP teaching as opposed to stand-alone NP teaching. Co-teaching dynamics appear to not adversely affect the cognitive or social congruence acquired during standalone teaching. By exploring student perceptions and knowledge gain, we provide some tentative evidence to suggest that, in our setting, the optimal number of NP teachers lies between 5–8 (in an 8-station practical), using teaching conditions that adopt the highest levels of organisational structure and time management. It is unlikely that the continued supplement of NP teachers has a linear relationship with positive learning or student experience outcomes. Although students find high levels of structure desirable, it may not be the most appropriate pedagogical approach to prepare them for the level of autonomy required in the clinical years of study.

## Data Availability

The datasets generated during and/or analysed during the current study are available from the corresponding author on reasonable request.

## Appendix B: A sample of representative quotes from participants in the focus group regarding all categories and codes

(i) **Benefits of NP Teaching**
  *“Near-Peer Teachers are happy to go deep [into the content], feel more welcoming, and you don’t feel bad asking to repeat themselves or asking them lots of questions... They’ve been in your shoes, so they don’t mind as much to repeat themselves.” ***– 3**
  *“They [NP teachers] were teaching us good ways of remembering things and going through loads of content... and then teaching us mnemonics… it was really useful.” ***– 3**
  *“They [NP teachers] get to practice teaching, which is an important skill because as a doctor, you will be teaching other colleagues or medical students... It’s quite nice for students to get that opportunity to practice that quite early.” ***– 2**
  *“[NP teaching] takes the weight off the staff because the NP teachers are there to support them” ***– 6**
(ii) **Credibility of NP Teaching**
  *“The NP teacher is just someone in the year above that you’re friendly with, probably play sports with or been out with. How much do you trust them with in terms of your education?” ***– 2**
(iii) **Implementing NP Teaching in Other Modules**
  *“I personally like to try and find out stuff myself. I actually look at it very closely and work out what it should be... I found it [exclusive faculty teaching] more useful than co-teaching [in NLM2 practical NP teaching sessions]” ***– 5**
  *“What people need to realize about NP teaching and teaching in general [is that] a lot of it you have to do it by yourself, as much as it’s great to have somebody there to show you... you’re going still have to come back and see for yourself” ***– 1**
  *“I also remember learning a lot of things incorrectly… someone had told me that was right. Then you ask a faculty teacher a few weeks later, and they’re like no, you’re completely wrong. Then having to unlearn that and then learn new things is so much more confusing than just being told this is what it is.” ***– 6**
(iv) **Importance of Faculty Involvement**
  *“[Co-teaching] is probably the best of both worlds, especially if you do have a more challenging question that the NP teachers are not sure because they can’t remember the details.” ***– 6**
  *“[NP teaching] can work in both classroom and anatomy lab environments as long as someone’s checked over the material and the teaching has been accredited essentially” ***– 2**
  *“At the end of the day, I also would like to get learning by world-renowned scientists who have their qualifications... we should be careful to completely dismiss the faculty” ***– 1**
